# Nutrient improvement for simultaneous production of exopolysaccharide and mycelial biomass by submerged cultivation of *Schizophyllum commune* AGMJ-1 using statistical optimization

**DOI:** 10.1007/s13205-012-0103-3

**Published:** 2012-11-24

**Authors:** Mayur Joshi, Harshad Patel, Shilpa Gupte, Akshaya Gupte

**Affiliations:** 1 Department of Microbiology, Natubhai V. Patel College of Pure and Applied Sciences, Vallabh Vidyanagar, 388 120 Gujarat India; 2 Department of Microbiology, VP and RPTP Science College, Vallabh Vidyanagar, 388 120 Gujarat India; 3 Ashok and Rita Patel Institute of Integrated Study and Research in Biotechnology and Allied Sciences, New Vallabh Vidyanagar, 388 121 Gujarat India

**Keywords:** Exopolysaccharide, Mycelial biomass, *Schizophyllum commune*, One factor at a time, Plackett–Burman design, Response surface methodology

## Abstract

Exopolysaccharides (EPS) of fungal origin have attracted special attention from researchers due to their multifarious applications in the food and pharmaceutical industries. In the present study, optimization of the process parameters for the production of exopolysaccharide by *Schizophyllum commune* AGMJ-1 was studied using one factor at a time (OFAT) method, Plackett–Burman design (PBD) and response surface methodology (RSM). OFAT method revealed xylose and yeast extract to be the most effective carbon and nitrogen sources and pH 5.3 as an optimum for maximum EPS production. Xylose, yeast extract and KCl were screened as statistically significant variables for EPS production using PBD. RSM based on the central composite design estimated that maximum EPS (4.26 g L^−1^), mycelial biomass (14 g L^−1^) and specific yield (0.45 g g^−1^) were obtained when concentration of xylose, yeast extract and KCl were set at 2.5 g % (w/v), 0.83 g % (w/v) and 6.53 mg % (w/v), respectively, in the production medium.

## Introduction

The increased demand of natural polymers for various industrial applications in recent years has led to a renewed interest in exopolysaccharide production by microorganisms. Many microorganisms have an ability to produce exopolysaccharide extracellularly either as soluble or insoluble polymers. Recently many polysaccharides and polysaccharide protein complexes have been isolated from marine algae, lichens and plants. Furthermore, most of them which have various physiological activities originated from fungi, especially from mushrooms (Ooi and Liu 1999; Yang et al. 2000; Cohen et al. 2002). Production of exopolysaccharide is widely distributed among fungi and most of the exopolysaccharide produced by them are highly hygroscopic *β*-glucan, suggesting that its production could be related with tolerance to desiccation; similarly to that observed and described in bacteria (Schinder-Keel et al. 2001; Selbmann et al. 2003). In white rot fungi, the production of exopolysaccharide could also be related to decay process of wood and protective layer (Vesentini et al. 2007). The exopolysaccharide consist of large variety of complex chemical structures, physiological functions and wide range of potential applications in various industrial areas as food engineering, biodegradable plastics, agronomy, pharmaceuticals, fuels and others. Therefore, these polymers are renewable resources that offer real and potential uses for humankind. The compounds of fungal origin have attracted special attentions from researchers due to their various pharmacological and biological activities, such as antitumor, antidiabetic, antimicrobial and immunostimulatory. Among these compounds, bioactive polysaccharides isolated from higher basidiomycota are the best known and appear to have strongest anticancer activity among mushroom-derived substances. Cultivation of mushrooms requires several weeks to complete fruiting body formation and thus delaying the production of the required compounds. However, submerged cultivation on fungi offers an advantage for the production of mycelial biomass and bioactive polysaccharides (Zhong and Tang 2004).

*Schizophyllum commune* is a species of basidiomycetes belonging to the schizophyllaceae family of order agaricales. It has been reported to be a filamentously growing fungus that produces exopolysaccharide (EPS) and secretes *β*-glucan as primary molecular structure. Sizophiran an antitumor polysaccharide from culture broth of *S. commune* reported by Shittu et al. (2005) served as an effective immunotherapeutic agent for cervical carcinoma. Fagade and Oyelade (2009) reported various polysaccharide antitumor agents such as hetero *β*-glucan and their complexes from the fruiting body, mycelia and culture broth of mushrooms. EPS production rate and productivity were found varying with environmental conditions and nutrient medium.

In the present investigation, the most effective pH, carbon and nitrogen sources for EPS production and mycelial biomass were studied using one factor at a time (OFAT) method. A Plackett–Burmann (PBD) design was then applied followed by response surface methodology (RSM) to optimize medium composition for maximal production of EPS and biomass. Fermentation conditions were found to have a positive effect on the production of metabolites and culture morphology.

## Materials and methods

### Organism and culture conditions for growth

Fungus was isolated in our laboratory from decayed wood in Anand, Gujarat, India and identified as *S. commune* AGMJ-1 (Gene Bank Accession No.-JQ023130). The organism was cultivated on malt extract (ME) agar plate containing: malt extract 2 %, agar 2.5 %. The culture was maintained at 4 °C.

### Culture process and EPS production

The shake flask cultivation was carried out in 250 ml Erlenmeyer flask containing 100 ml of sterilized (121 °C, 15 lbs for 15 min) EI-Naghy medium (El-Naghy et al. 1976). The media consisted following components (% w/v): glucose 2.0; NaNO_3_ 0.2; yeast extract 0.05; KH_2_PO_4_ 0.1; MgSO_4_∙7H_2_O 0.05 KCl 0.05 and pH 5.3. Agar discs were punched out with a sterilized cup borer from the edge of 7-day-old fungal culture grown on ME agar plate. The flasks containing fermentation medium were inoculated with four agar discs of 7 mm diameter and incubated on a rotatory incubator shaker (140 rpm) at 30 °C for 14 days. Growth kinetics of *S. commune* AGMJ-1 isolated in our laboratory was studied in shake flasks.

### Determination of exopolysaccharide and mycelial biomass

Samples were harvested at different time interval from the shake flasks. The fermentation broth was centrifuged at 4 °C at 8,000 rpm for 10 min to separate fungal biomass, and the resulting supernatant filtered then through Whatman filter paper no. 1. The mycelial dry weight was measured after repeated washing of mycelial pellets with distilled water and dried at 70 °C in hot-air oven to a constant weight. For the determination of exopolysaccharide, equal volume of isopropanol was mixed with mycelia-free culture filtrate, stirred vigorously and left overnight at 4 °C for precipitation. The resultant precipitates obtained after 24 h were separated by centrifugation (8,000 rpm for 10 min.).

The precipitates collected (crude EPS fraction) was washed twice with isopropanol and dried to a constant weight at 70 °C. The yield of mycelial biomass and EPS were expressed as the gram per liter of the culture medium (g L^−1^). The residual sugar content of the culture filtrate was determined by 3,5-dinitrosalicylic acid method (DNSA) method using glucose as standard (Miller 1959).

### One factor at a time (OFAT) method

In each experiment, one factor was varied by holding other factors constant. Different carbon sources, nitrogen sources and pH were initially studied by employing single factor experiment.

### Plackett–Burman design

Plackett–Burman design is a most saturated design (Plackett and Burman 1946). A saturated design is a one in which the number of design points are equal to one more than the number of factor of effects to be estimated and such design allows unbiased estimation of all main effects with smallest possible variance. Moreover, the design is orthogonal in nature implying that the effect of each variable worked and pure in nature and not confound to interaction among variables (Simpson et al. 2001). In the present investigation, it was used for the study of key factors prior to optimization. A total of six variables were screened including xylose, yeast extract, MgSO_4_∙7H_2_O, KH_2_PO_4_, KCl and inoculum size (Table 1). Table 2 shows the Plackett–Burman experimental design for 12 trials with two levels of concentrations for each variable and the corresponding exopolysaccharide and mycelial biomass in terms of (g L^−1^) of the culture medium. The variables *X*_1_–*X*_6_ represents the experimental variables, whereas *D*_1_–*D*_5_ represents the dummy variables. Table 3 shows the probability value of various process parameters selected for screening in PBD.

**Table 1:** Variables showing process parameters used in Plackett–Burman design

Variables	Medium components	Positive values (% w/v)	Negative values (% w/v)
X_1_	Xylose	4.0	0.4
X_2_	Yeast extract	1.0	0.1
X_3_	MgSO_4_∙7H_2_O	0.5	0.05
X_4_	KCl	0.1	0.01
X_5_	KH_2_PO_4_	0.1	0.01
X_6_	Inoculum size	0.057	0.019

**Table 2:** Plackett–Burman design matrix of six process variables (*X*_1_–*X*_6_) and five dummy variables (*D*_1_–*D*_5_) along with the observed responses (EPS and mycelial biomass)

Run no.	*X*_1_	*X*_2_	*X*_3_	*X*_4_	*X*_5_	*X*_6_	*D*_1_	*D*_2_	*D*_3_	*D*_4_	*D*_5_	EPS (g L^−1^)	Mycelial biomass (g L^−1^)
1	**+**	**+**	−	**+**	**+**	**+**	−	−	−	**+**	−	3.53	20.54
2	−	**+**	**+**	−	**+**	**+**	**+**	−	−	−	**+**	1.36	3.81
3	**+**	−	**+**	**+**	−	**+**	**+**	**+**	−	−	−	1.80	11.81
4	−	**+**	−	**+**	**+**	−	**+**	**+**	**+**	−	−	0.43	2.40
5	−	−	**+**	−	**+**	**+**	−	**+**	**+**	**+**	−	0.88	3.55
6	−	−	−	**+**	−	**+**	**+**	−	**+**	**+**	**+**	0.18	3.60
7	**+**	−	−	−	**+**	−	**+**	**+**	−	**+**	**+**	3.25	9.22
8	**+**	**+**	−	−	−	**+**	−	**+**	**+**	−	**+**	3.64	19.85
9	**+**	**+**	**+**	−	−	−	**+**	−	**+**	**+**	−	4.48	17.10
10	−	**+**	**+**	**+**	−	−	−	**+**	−	**+**	**+**	0.60	2.84
11	**+**	−	**+**	**+**	**+**	−	−	−	**+**	−	**+**	2.11	11.20
12	−	−	−	−	−	−	−	−	−	−	−	0.38	2.42

**Table 3:** Statistical analysis of process parameters in relation to EPS and mycelial biomass

Factors	Fermentation parameters	EPS (g L^−1^)				Mycelial biomass (g L^−1^)			
		Effect *E*_(*xi*)_	SE	*t*_(*xi*)_	*P* value	Effect *E*_(*xi*)_	SE	*t*_(*xi*)_	*P* value
*X*1	Xylose	14.98	1.63	9.218	0.00025	71.1	8.58	8.286	0.0094
*X*2	Yeast extract	5.44		3.347	0.020	24.74		2.883	0.034
*X*3	MgSO_4_∙7H_2_O	−0.18		−0.1107	0.916	−7.72		0.899	0.4
*X*4	KCl	−5.34		−3.286	0.021	−3.56		0.414	0.69
*X*5	KH_2_PO_4_	0.48		0.295	0.770	−6.90		0.803	0.45
*X*6	Inoculum	0.14		0.086	0.930	17.98		2.09	0.09

### Response surface methodology (RSM)

The objective of this experiment is to develop an experimental model of the process and to obtain more precise estimate of the optimization. The approach to an optimization process is referred to as (RSM) and the second design is central composite design (CCD), one of the most important design used in the process optimization method (Montgomery 1997). RSM was used in the present study, as the conventional method of optimization OFAT is laborious, time consuming and incomplete. RSM using CCD as a collection of mathematical and statistical techniques which was applied for the modeling and analysis of the problems in which a response of interest is influenced by several variables and the objective is to optimize the response (Teoh and Mat Don 2010; Zinatizadeh et al. 2007). The behavior of the system was demonstrated by the following quadratic equation.1where *Y* is the predicted response, *β*_0_ is a constant, *β*_*i*_ is the linear coefficient, *β*_*ii*_ is squared coefficient, *β*_*ij*_ is the cross product coefficient, *x*_*i*_ is the dimensionless coded value of (*X*_*i*_). The above equation was solved using the software Design-Expert (Version 7.0.2, State ease Inc., USA). A 2^5^ factorial design with five replicates at the center point with a total number of 20 trials were employed. EPS production and mycelial biomass were expressed as a gram per liter of culture of medium (g L^−1^) and specific yield as gram EPS per gram of dry mycelial biomass (g g^−1^).

## Results and discussion

### Screening of process parameters using OFAT method

**Fig. 1:**
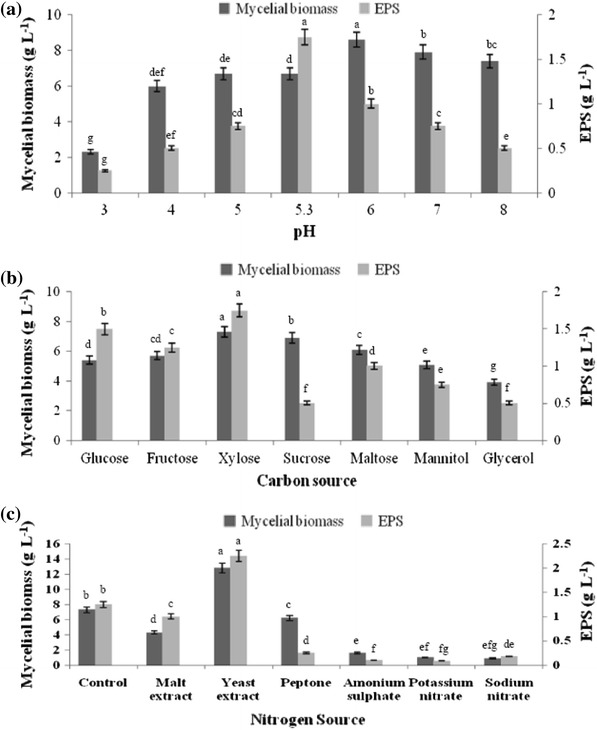
Effect of **a** pH, **b** carbon source, **c** nitrogen source on EPS production and mycelial biomass. Each sample was assayed in triplicate for all treatments. Standard deviation around the mean is represented by the *bars*. ANOVA was performed and differences were considered statistically different at *P* < 0.05

The pH of the culture medium is critical for the production of EPS. In our experiment, we examined the effect of initial pH on the production of EPS and mycelial biomass. The initial pH of the culture medium was adjusted in the range of pH 3–8. The results depict that maximum mycelial biomass (8.6 g L^−1^) was obtained at pH 6.0, whereas at the same pH production of EPS was found to be less than the EPS produced at pH 5.3 (Fig. 1a). A gradual increase in EPS production was observed from pH 3 and reached maximum of 1.75 g L^−1^ at pH 5.3. The results obtained confirm that acidic conditions are necessary for the growth of higher fungi. Shu and Lung (2003) reported that the production of EPS and mycelial biomass could not be correlated well. This may be due to the effect of pH which has a profound effect on mycelial morphology, mycelial growth, broth rheology and metabolite production. Lee et al. (1999) reported that maximum production of mycelial biomass and EPS in *Ganoderma lucidum* were achieved at pH of 3 and 6, respectively. The results obtained by us are thus in accordance with other reports (Hwang et al. 2007; Malinowska et al. 2009).

Lee et al. (2004) and Feng et al. (2010) reported that the type and amount of carbon source have an influence on the biosynthesis of secondary metabolites. The influence of the carbon sources for mycelial biomass and EPS production was studied in media containing different carbohydrates [monosaccharides, disaccharides and sugar alcohols at 2 g % (w/v)]. Figure 1b shows the effect of various carbon sources amongst which xylose significantly promoted the mycelial growth as well as the EPS production. It was found that the monosaccharides yielded higher production of EPS then the disaccharides and sugar alcohols. Similar results were also obtained by Kim et al. (2006) using *Ganoderma recinaceum* DG-6556. Maximum yield of EPS was obtained when xylose was used as a carbon source followed by glucose and fructose. Furthermore, the results obtained also indicate that the mycelial growth was related to EPS production in case of xylose as carbon source when compared with other disaccharides and sugar alcohols used. Similar observation was also reported by Guo et al. (2009) who stated that suitable concentration for mycelial biomass and EPS production from 20 to 50 g L^−1^. Choi et al. (2011) reported lactose as choice of carbon source for EPS production using *Mycoleptodonoides aitchisonii* and Lin (2011) reported fructose as suitable carbon source for EPS production using *Grifola frondosa*.

Nitrogen is one of the key nutrients for fungus in the synthesis of enzymes involved in the production of both primary and secondary metabolites. To investigate the effect of nitrogen source, both organic and inorganic nitrogen sources were examined (Fig. 1c). Amongst various nitrogen sources used, yeast extract (organic nitrogen source) was found to be the most significant for influencing mycelial growth and EPS production, while the presence of inorganic nitrogen sources were less effective and showed poor biomass and EPS production. This can be attributed that nitrate ions have an inhibitory effect on growth, and sulfate ions are larger radicals which may be difficult to traverse across the fungal membrane, where it can promote growth (Griffin 1994). Yeast extract was found be the best nitrogen source in our study and is often used to provide necessary growth factors and serves as a complex nitrogen source; thus, giving a higher EPS production and mycelial biomass. Yeast extract as a choice of nitrogen source have been reported by various researchers (Lin 2011; Park et al. 2001; Lee et al. 2004).

### Statistical optimization using PBD and RSM

Plackett–Burman results indicated xylose, yeast extract and KCl had a significant influence on EPS production, and were further selected for optimization of their concentration by the subsequent CCD.

The experimental design performed by the RSM method is based on the mathematical techniques that enable us to investigate the interactions between variables of the medium components. The CCD was used to determine the optimal concentration (level) of the medium components. A total of 20 experiments with three variables (components of the medium) and five coded levels (five different concentrations) were performed. Based on the results obtained from the PBD, we selected three variables; namely, xylose, yeast extract and KCl. Amongst them xylose and yeast extract showed positive influence on both EPS production and mycelial biomass. Thus, increasing concentrations of xylose and yeast extract resulted in higher mycelial growth and EPS production. Although KCl had negative influence on EPS production hence lower concentration of KCl resulted in higher EPS production. The other components of the production medium; namely, MgSO_4_∙7H_2_O, KH_2_PO_4_ and inoculum size were found to be insignificant, so their concentrations were set at their middle level in CCD. Table 4 represents the experimental design matrix for CCD along with the experimental results of predicted responses for EPS, mycelial biomass and specific yield, respectively. The experimental values for the regression coefficient were obtained by quadratic polynomial equation, where only significant coefficients (*P* < 0.05) were considered. The smaller *P* values indicate the higher significance of the corresponding coefficient. The insignificant coefficients were not omitted from the equations, since it was a hierarchical model.

**Table 4:** Central composite design (CCD) matrix of independent variables and the corresponding experimental and predicted values for EPS, mycelial biomass and specific yield

Run no.	Factors						Responses					
	A: Xylose (g % w/v)		B: Yeast extract (g % w/v)		C: KCl (mg % w/v)		EPS (g L^−1^)		Mycelial biomass (g L^−1^)		Specific yield (g g^−1^)	
	Coded value	Actual value	Coded value	Actual value	Coded value	Actual value	Observed value	Predicted value	Observed value	Predicted value	Observed value	Predicted value
1	−1	2.50	−1	0.83	−1	6.61	3.54	3.56	10.21	9.68	0.35	0.35
2	0	3.30	0	1.10	−2	6.0	4.01	4.15	15.28	15.1	0.26	0.28
3	0	3.30	+2	1.55	0	7.50	2.32	2.35	13.27	13.3	0.18	0.19
4	+1	4.10	+1	1.37	+1	8.39	2.90	3.02	15.10	15.4	0.19	0.19
5	+1	4.10	+1	1.37	−1	6.61	2.80	2.81	13.30	12.9	0.21	0.22
6	−1	2.50	+1	1.37	+1	8.39	2.67	2.76	14.97	14.8	0.18	0.18
7	−1	2.50	−1	0.83	+1	8.39	2.53	2.52	13.88	14.0	0.19	0.18
8	+2	4.65	0	1.10	0	7.50	2.43	2.54	12.21	12.6	0.2	0.21
9	0	3.30	0	1.10	+2	9.0	2.0	2.03	8.73	9.18	0.23	0.24
10	+1	4.10	−1	0.83	+1	8.39	2.76	2.55	12.91	12.6	0.21	0.21
11	−1	2.50	+1	1.37	−1	6.61	4.21	4.12	14.0	14.7	0.3	0.28
12	0	3.30	0	1.10	0	7.50	3.0	2.92	16.43	15.9	0.18	0.17
13	0	3.30	−2	0.65	0	7.50	3.70	3.58	13.64	13.8	0.27	0.27
14	0	3.30	0	1.10	0	7.50	2.60	2.54	14.19	14.2	0.18	0.18
15	0	3.30	0	1.10	0	7.50	3.13	2.82	12.20	12.1	0.26	0.23
16	+1	4.10	−1	0.83	−1	6.61	3.0	2.82	11.95	12.1	0.25	0.23
17	−2	1.95	0	1.10	0	7.50	2.70	2.82	12.15	12.1	0.22	0.23
18	0	3.30	0	1.10	0	7.50	2.82	2.82	11.81	12.1	0.24	0.23
19	0	3.30	0	1.10	0	7.50	2.68	2.82	12.54	12.1	0.21	0.23
20	0	3.30	0	1.10	0	7.50	2.58	2.82	11.98	12.1	0.22	0.23

The predicted responses *Y* for the EPS, mycelial biomass and specific yield were obtained as follows:234where *Y*_1_ is the EPS (g L^−1^), *Y*_2_ is mycelial biomass (g L^−1^) and *Y*_3_ is the specific yield (g g^−1^) and A, B, C are coded values of the independent variables (xylose, yeast extract and KCl, respectively). The statistical significance of the quadratic model for the experimental responses was evaluated by the analysis of variance (ANOVA). According to the ANOVA results (Table 5), the model was significant with an *F* test of a very low probability value (*P* > *F*) <0.0001 for the EPS, mycelial biomass and specific yield, respectively. The goodness of fit for the model was expressed by the coefficient of determination *R*^2^, and the values were found to be 0.904, 0.963 and 0.905 for EPS, mycelial biomass and specific yield, respectively. The values of *R*^2^ indicate that the experimental values were significantly in agreement with the predicted values and also suggested that the model is suitable and practicable. The lack of fit *F* values 0.62 and 0.65 for EPS and specific yield, respectively, were not significantly relative to pure error. These large values could occur due to noise. Whereas in case of mycelial biomass, the lack of fit *F* value of 5.84 is significant, which indicates that there was only 3.76 % chance that this value could occur due to noise. The significant lack of fit is bad because we want the model to fit. The purpose of statistical analysis is to determine which experimental factors generate signals, which are large in comparison to noise. The adequate precision value measures signal to noise ratio and ratio greater than 4.0 is desirable. In the present study, the value of this ratio was higher for mycelial biomass and suggested that the polynomial quadratic model can be used to navigate the design space and further optimization. The 3-D surface plots and their respective contour plots illustrate the response over a region of interesting factor levels, the relationship between the response and experimental levels of each variable and the type of interactions between the test variables in order to deduce the optimal composition of the culture medium. Each plot depicts the effect of two independent variables varying within the experimental range of EPS, mycelial biomass and specific yield, while the other variable was fixed at its optimal value. In contrast to the circular shape contour plots, the elliptical nature of the curves indicates significant mutual interactions between variables.

**Table 5:** Analysis of variance (ANOVA) for the experimental results of the CCD

Factor	EPS (g L^−1^)			Mycelial biomass (g L^−1^)			Specific yield (g g^−1^)		
	Sum of squares	*F* value	*P* value (*P* > *F*)	Sum of squares	*F* value	*P* value (*P* > *F*)	Sum of squares	*F* value	*P* value (*P* > *F*)
Model	5.5815	17.43	<0.0001	58.5	29.1	<0.0001	0.0341	20.7	<0.0001
A	0.3223	9.057	0.0131	14.2	63.5	<0.0001	0.0009	3.43	0.0870
B	1.7402	48.9	<0.0001	1.68	7.5	0.0209	0.0142	51.9	<0.0001
C	1.2855	36.12	0.0001	0.15	0.66	0.4339	0.0089	32.4	<0.0001
AB	0.0024	0.069	0.7984	5.41	24.2	0.0006	0.0025	9.2	0.0096
AC	0.2048	5.754	0.0374	5.95	26.6	0.0004	0.0002	0.88	0.3644
BC	0.4141	11.63	0.0066	3.20	14.3	0.0036	0.0073	26.7	0.0002
A^2^	0.5119	14.38	0.0035	2.61	11.7	0.0066			
B^2^	0.8624	24.23	0.0006	18.4	82	<0.0001			
C^2^	0.1011	2.841	0.1228	6.45	28.8	0.0003			

*A* xylose, *B* yeast extract, *C* KCl

**Fig. 2:**
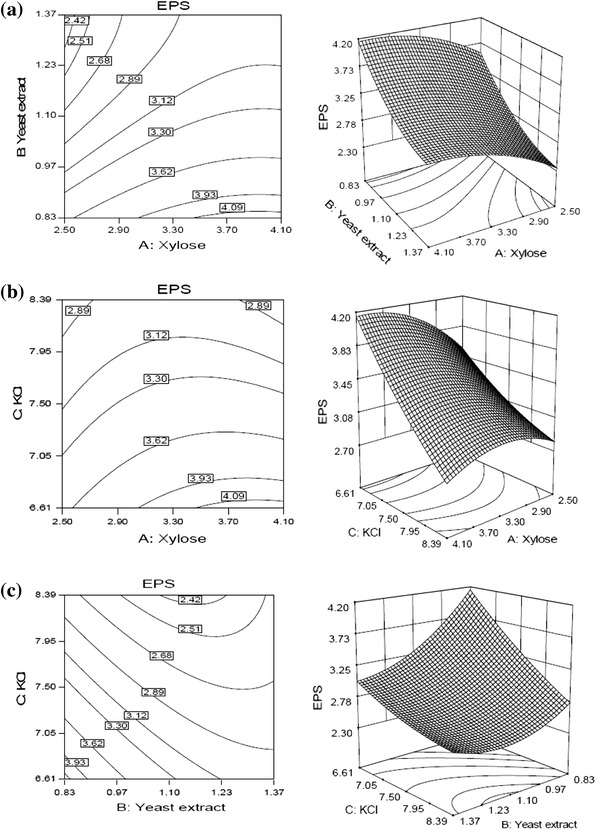
Response surfaces and corresponding contour plots for EPS production by *S. commune* AGMJ-1 as function of **a** xylose and yeast extract, **b** xylose and KCl, **c** yeast extract and KCl

Response surface and contour plots (Fig. 2a) show the effect of xylose and yeast extract on the EPS production at specific hold value of KCl (6.61 mg % w/v). EPS production increased with increasing concentration of xylose and decreasing concentration of yeast extract. Maximum yield of EPS obtained at higher value of xylose and lower value of yeast extract. The same trend of results was also observed in 3-D surface curves (Fig. 2b), which shows the effect of xylose and yeast extract on EPS production at specific hold value of yeast extract (0.83 g % w/v). The production EPS was maximum at higher value of xylose and lower value of KCl.

It is possible to observe in Fig. 2c, the effect of yeast extract and KCl on EPS production at specific hold value of xylose (3.97 g % w/v). The production of EPS increased with decreasing the concentration of both yeast extract and KCl and reaches to the maximum at their lower values. There was no significant alteration in production of EPS observed with increasing yeast extract concentration at higher value of KCl. However, at lower value of KCl, increasing concentration of yeast extract results in decreased production of EPS.

**Fig. 3:**
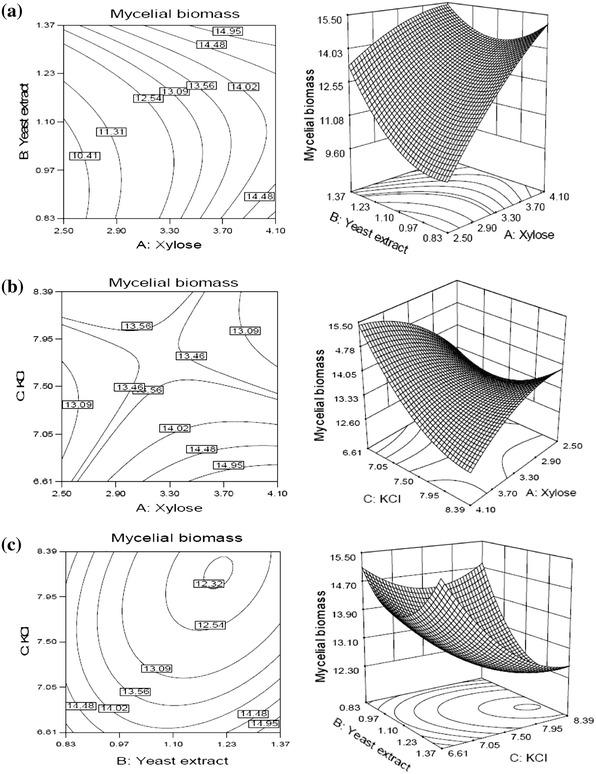
Response surfaces and corresponding contour plots for mycelial biomass production by *S. commune* AGMJ-1 as function of **a** xylose and yeast extract, **b** xylose and KCl, **c** yeast extract and KCl

Figure 3a explains the effect of xylose and yeast extract on mycelial biomass at specific hold value of KCl (6.52 mg % w/v). It indicates that the mycelial biomass was maximum at higher values of both the variables. At higher value of yeast extract, increasing concentrations of xylose results in increased mycelial biomass. Although at higher value of xylose, the mycelial biomass starts to decrease with increasing yeast extract concentration up to certain level and then again increased with further increase in yeast extract concentration.

Figure 3b depicts the effect of xylose and KCl on mycelial biomass at specific hold value of yeast extract (1.37 g % w/v). The maximum yield of mycelial biomass achieved at lower value of KCl and higher value of xylose. At lower level of KCl, increasing concentration of xylose results in increased production of mycelial biomass and reaches to the maximum. However, at lower value of xylose, biomass production decreased not much with increasing KCl concentration up to certain level thereafter, rise in biomass production observed with further increasing KCl concentration.

The 3-D surface plots (Fig. 3c) elucidate the effect of yeast extract and KCl on mycelial biomass at specific hold value of xylose (4.10 g % w/v). The maximum mycelial biomass was observed at lower value of KCl and higher value of yeast extract. At lower value of KCl, decreasing concentration of yeast extract resulted in gradual decrease in mycelial biomass up to certain level, thereafter, mycelial biomass increased and reaches near to the maximum. The same trend of results was observed at lower value of yeast extract with decreasing concentration of KCl.

**Fig. 4:**
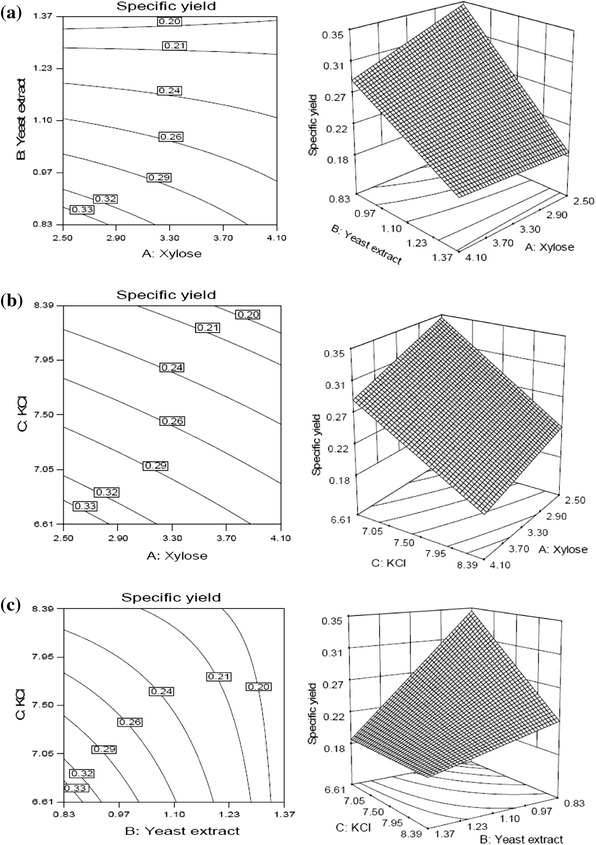
Response surfaces and corresponding contour plots for specific yield by *S. commune* AGMJ-1 as function of **a** xylose and yeast extract, **b** xylose and KCl, **c** yeast extract and KCl

The 3-D surface plots and contour plots (Fig. 4a) illustrate the effect of xylose and yeast extract on specific yield at specific hold value of KCl (6.53 mg % w/v). The specific yield was increased with decreasing xylose and yeast extract concentrations and yielded maximum at lower values of both the variables. At higher value of xylose, decreasing concentration of yeast extract results in increased specific yield. However, there was no significant change was observed in specific yield at higher value of yeast extract with decreasing xylose concentration. Similar trend of results was observed in Fig. 4b, which shows the effect of xylose and KCl on specific yield at specific hold value of yeast extract (0.83 g % w/v). The maximum specific yield was obtained at lower values of both xylose and KCl.

The 3-D surface plot (Fig. 4c) shows the effect of yeast extract and KCl on specific yield at specific hold value of xylose (2.5 g % w/v). The specific yield increased by decreasing the concentration of both yeast extract and KCl and reached to the maximum at lower values of both the variables. The specific yield was almost constant at higher value of yeast extract with increasing concentration of KCl. However, at higher value of KCl, decreasing concentration of yeast extract resulted in little increase in specific yield.

The data obtained from the 3-D surface plots and contour plots and the equations obtained from the multiple regression analysis, we can determine the optimal concentration of the medium components. The model predicts that the EPS production (4.16 g L^−1^) is located at the actual values (w/v): xylose 3.97 g %, yeast extract 0.83 g %, and KCl 6.61 mg %. The predicted mycelial biomass (15.7 g L^−1^) corresponding to the actual concentration (w/v): xylose 4.10 g %, yeast extract 1.37 g %, and KCl 6.52 mg %, while specific yield reaches to its maximum value of (0.35 g g^−1^) at the actual concentration (w/v): xylose 2.5 g %, yeast extract 0.83 g % and KCl 6.52 mg %.

To determine the best possible combination for each response simultaneously, the graphical optimization of the overall desirability function was performed. The predicted optimal values for the variables were as follows (w/v): xylose 2.5 g %, yeast extract 0.83 g %, and KCl 6.53 mg %, whereas, the predicted responses were EPS 3.61 g L^−1^, mycelial biomass 9.66 g L^−1^ and specific yield 0.35 g g^−1^, respectively.

### Validation of the experiments

**Fig. 5:**
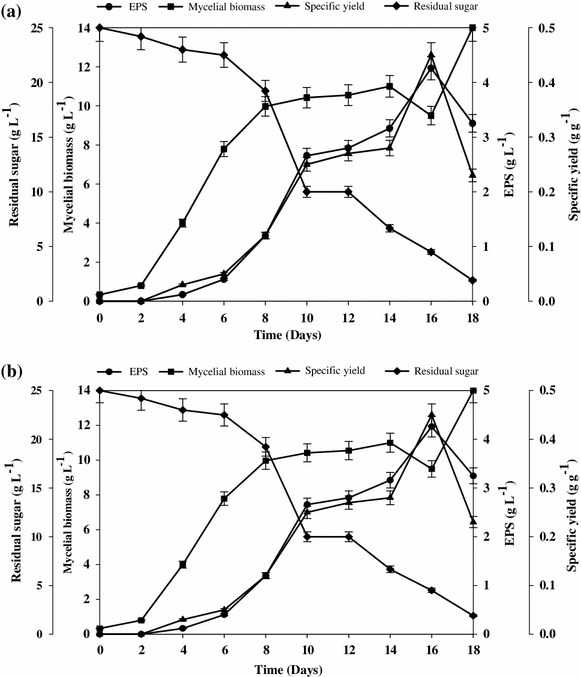
Time course study of *S. commune* AGMJ-1 **a** unoptimized medium, **b** optimized medium for EPS, mycelial biomass, specific yield and residual sugar

A typical time course between statistically optimized medium and unoptimized medium was compared for 18 days in shake flask conditions (Fig. 5a, b). The amount of EPS produced, mycelial biomass, specific yield and residual sugar were monitored. It was found that under statistically optimized medium increase in EPS (4.26 g L^−1^) with 4.0-fold, mycelial biomass (14 g L^−1^) with 1.5-fold and specific yield (0.45 g g^−1^) with 2.5-fold were obtained as compared to the unoptimized medium. Furthermore, an interesting observation was made related to the formation of insoluble gel when the culture filtrate was subjected to freezing before polysaccharide precipitation. This peculiar characteristic could aid in polymer separation and reduce the utilization of solvent for precipitation and thus increasing the process viability.

## Conclusion

*Schizophyllum commune* AGMJ-1 is known to be a potential producer of secondary metabolites. The OFAT method was carried out to determine the effect of carbon source, nitrogen source and pH for mycelial biomass and EPS production. The methodology of Plackett–Burman was found to be useful for determination of relevant variables for further optimization. The use of this technique helped in finding the important medium components that have significant effect on EPS production by *S. commune* AGMJ-1. The experimental data fitted well with the model predicted values. In conclusion, the methodology of PBD and RSM using CCD has proved to be very effective for the optimization of the exopolysaccharide production.
